# Supplementary material for: Index microvascular resistance (IMR) in heart transplant patients (IMR-HT study): Study protocol
Source: PLoS One. 2025 May 16;20(5):e0315053. doi: 10.1371/journal.pone.0315053 (PMC12084034; doi:10.1371/journal.pone.0315053)
Supplement: S1 File — (DOCX) [file pone.0315053.s001.docx]

**Index microvascular resistance (IMR)-guided management of heart transplantation**

**Protocol**

Version draft - November 13, 2024

| Sponsors: | Interventional Cardiology Department Miguel Servet University Hospital. Zaragoza, Spain |
| --- | --- |
| Protocol contributors: | Ainhoa Perez Guerrero, (Clínico Lozano Blesa University Hospital. Zaragoza. Spain.)  Teresa Blasco Peiro, (Miguel Servet University Hospital. Zaragoza. Spain.)  Salvatore Brugaletta, (Hospital Clínic, Cardiovascular Clinic Institute, Institut d’Investigacions Biomèdiques August ) |
| Trial identifiers: | NCT 06656065 |

**SIGNATURE PAGE**

**REVISION HISTORY**

| Version | Date | Amendment Text | Description |
| --- | --- | --- | --- |

**Table of Contents**

[1. TRIAL SUMMARY](#_Toc1)

[2. INTRODUCTION](#_Toc2)

[2.1. Background and rationale](#_Toc3)

[2.2. Objectives](#_Toc4)

[2.3. Trial design](#_Toc5)

[3. METHODS](#_Toc6)

[3.1. Study setting](#_Toc7)

[3.2. Eligibility criteria](#_Toc8)

[3.3. Interventions](#_Toc9)

[3.3.1. Intervention description](#_Toc10)

[3.3.2. Modifications](#_Toc11)

[3.3.3. Adherence](#_Toc12)

[3.3.4. Concomitant care](#_Toc13)

[3.4. Outcomes](#_Toc14)

[3.5. Participant timeline](#_Toc15)

[3.6. Sample size](#_Toc16)

[3.7. Recruitment](#_Toc17)

[3.8. Allocation](#_Toc18)

[3.9. Blinding (masking)](#_Toc19)

[3.9.1. Blinding mechanism](#_Toc20)

[3.9.2. Emergency unblinding](#_Toc21)

[3.10. Data collection](#_Toc22)

[3.10.1. Trial procedures and evaluations](#_Toc23)

[3.10.2. Retention](#_Toc24)

[3.11. Data management](#_Toc25)

[3.12. Statistical methods](#_Toc26)

[3.12.1. Outcomes](#_Toc27)

[3.12.2. Additional analyses](#_Toc28)

[3.12.3. Analysis population and missing data](#_Toc29)

[3.13. Data monitoring](#_Toc30)

[3.14. Safety/harms](#_Toc31)

[3.15. Auditing](#_Toc32)

[4. ETHICS AND DISSEMINATION](#_Toc33)

[4.1. Research ethics approval](#_Toc34)

[4.2. Protocol amendments](#_Toc35)

[4.3. Informed consent process](#_Toc36)

[4.4. Confidentiality](#_Toc37)

[4.5. Declaration of interests](#_Toc38)

[4.6. Access to data](#_Toc39)

[4.7. Dissemination policy](#_Toc40)

[4.7.1. Trial results](#_Toc41)

[4.7.2. Authorship](#_Toc42)

[4.7.3. Reproducible research](#_Toc43)

[5. STUDY ADMINISTRATION](#_Toc44)

[5.1. Key contacts](#_Toc45)

[5.2. Funders](#_Toc46)

[5.3. Roles and responsibilities](#_Toc47)

[5.3.1. Protocol contributors](#_Toc48)

[5.3.2. Sponsor and funder](#_Toc49)

[5.3.3. Trial committees](#_Toc50)

[6. APPENDICES](#_Toc51)

[6.1. Informed consent materials](#_Toc52)

# 1. TRIAL SUMMARY

World Health Organization Registration Data Set

| Title | Index microvascular resistance (IMR)-guided management of heart transplantation |
| --- | --- |
| Primary registry and trial identifying number | ClinicalTrials.gov  NCT 06656065 |
| Secondary identifying numbers |  |
| Sources of monetary or material support | No funding. |
| Primary sponsor | Interventional Cardiology Department Miguel Servet University Hospital. Zaragoza, Spain |
| Secondary sponsors (if any) |  |
| Central contact | Georgina Fuertes Ferre  Interventional Cardiology Department Miguel Servet University Hospital. Zaragoza, Spain  0034 646 323 423  georginaff@hotmail.com |
| Study officials/Investigators |  |
| Brief title | IMR-HT study |
| Acronym | IMR-HT study |
| Countries of recruitment | Spain |
| Condition(s) or focus of study |  |
| Interventions |  |
| Key eligibility criteria | Age eligibility: 18 years or older  Sex eligibility: Both  Accepts healthy volunteers: No |
| Study design | Study type: Interventional trial  Allocation: Non-randomized  Intervention model: Single group  Primary purpose: Diagnostic  Phase: N/A |
| Masking | None |
| Date of enrollment | March 25, 2024 |
| Target sample size | 100 |
| Recruitment status | Recruiting |
| Primary outcomes |  |
| Secondary outcomes |  |

# 2. INTRODUCTION

## 2.1. Background and rationale

Acute allograft rejection (AAR) is an important cause of morbi-mortality  after heart transplant (HT), particularly within the first year. Advances in immunosuppression, donor heart evaluation, surgical techniques, and post-transplantation care have led to a gradual reduction in AAR and improved survival after HT over time. Endomyocardial biopsy (EMB) is the gold standard method to guide post-HT treatment, as it represents the best tool to identify rejection in orthotopic HT. However, it is usually repeated up to 5 times during the first year - with some variations depending on each center protocol - and it is potentially associated with serious complications.

Several studies have presented the association between AAR, micro-vasculopathy and cardiac allograft epicardial vasculopathy (CAV).  Index of microcirculatory resistance (IMR) measured early after heart transplantation has been significantly associated with the risk of acute cellular rejection (ACR), and patients with IMR≥15 have higher risk of AAR during 2 years follow-up.

The aim of our study is to evaluate if the use of IMR measured at baseline may be useful in guiding care of patients after HT.

## 2.2. Objectives

| **Primary endpoint** |
| --- |
| Evaluate IMR values and number of EMB performed in the first year after HT. |
| **Secondary endpoints** |
| Analyze the relationship between acute cellular rejection and IMR.  Analyze the relationship between cardiovascular and all-cause mortality and IMR.  Analyze the relationship between graft vasculopathy and IMR.  Analyze the relationship between the variation in the IMR value in the first two months and one year and cardiovascular events.  Analyze the relationship between a combined objective of ACR, heart failure, re-transplantation, cardiovascular mortality, all-cause mortality and IMR.  Assessment of immunosuppressive treatment after the IMR results. |

## 2.3. Trial design

The IMR-HT study is a multicenter, prospective study aimed to guide post-HT management based on IMR. It will enroll consecutive eligible patients who undergo HT in each participation center. Patients undergoing HT will be screened for enrollment. Eligible patients will be informed about the study and will have to provide informed consent prior to being included. The recruitment period of the study is two years.

# 3. METHODS

## 3.1. Study setting

All the studdy settings are spanish academic hospitals:

- Miguel Servet University Hospital.

- La Fe University Hospital.

- Bellvitge University Hospital.

- Asturias Central University Hospital.

- 12 Octubre University Hospital.

- Reina Sofía de Córdoba University Hospital.

- Virgen de la Arrixaca University Hospital.

- Virgen del Rocío University Hospital.

## 3.2. Eligibility criteria

***Inclusion criteria***

- Heart transplant patients >18 years.
- Patients who have received and signed informed consent.

***Exclusion criteria***

- Patients with hemodynamic instability after HT, including cardiogenic shock or severe coagulopathy.
- Patients with acute cellular rejection before intracoronary physiological assessment.
- Patients with bronchial asthma or bronchopathy with a positive bronchodilation test, which contraindicates the use of adenosine.
- Patients with epicardial coronary lesions with a resting physiological index ≤0.89 or ≤0.80 at hyperemia.
- Patients unlikely to cooperate or with inability or unwillingness to give informed consent.

## 3.3. Interventions

### 3.3.1. Intervention description

Based on IMR results, patient clinical status and the other complementary tests:

- If the IMR is less than 15, the number of biopsies could be reduced or kept the same. No immunosuppressive therapy changes would be made.

- If the IMR is 15 or greater, number of biopsies would be performed as usual per protocol. Immunosuppressive therapy could be intensified or maintained the same.

### 3.3.2. Modifications

N/A

### 3.3.3. Adherence

N/A

### 3.3.4. Concomitant care

N/A

## 3.4. Outcomes

- The **primary outcome** of the study is to evaluate IMR values and number of EMB performed in the first year after HT: Depending on IMR values obtained in the first three months, clinical status and the other complementary tests the physician will be able to modify the number of biopsies established in each center protocol.If the IMR is less than 15, the number of biopsies could be reduced or kept the same. If the IMR is 15 or greater,number of biopsies would be performed as usual per protocol.

**- Secondary outcomes:**

-IMR variation between first three months after HT and one year: Comparison between the values of index of microvascular resistance (IMR) measured by bolus thermodilution technique between the first and 3rd month after HT and one year in the invasive physiological study. IMR may be expressed as mmHg·s.

-Acute cellular rejection: A ≥2R degree according to the 2010 ISHLT system. Two or more focal infiltration points associated with myocyte injury in EMB. Diffuse infiltration with multi-focal myocyte injury with/without oedema, hemorrhage or vasculitis.Time frame: One year.

-Cardiac allograft vasculopathy: Accelerated fibroproliferative process characterized by diffuse, concentric and longitudinal thickening of the intima of the vascular tree of the graft, affecting everything from the major epicardial arteries to the coronary microvasculature.Time frame: 5 years.

-Cardiovascular mortality:Caused by cardiovascular disease or unknown death. Time frame: 5 years.

-Heart failure: Diastolic or systolic heart failure after heart transplant. Time Frame: Five years.

-Inmunosupressive therapy: Changes in any of the inmunosuppresive agents pre-established in each center heart transplant protocol. Time Frame: Five years

## 3.5. Participant timeline

Shedule of enrolment, interventions and assessments

| **TIMEPOINT (state unit)** |  |  |  |  |  |
| --- | --- | --- | --- | --- | --- |
| **VISIT NUMBER:** |  |  |  |  |  |
| **ENROLLMENT:** |  |  |  |  |  |
| Eligibility screen | X |  |  |  |  |
| Informed consent | X |  |  |  |  |
| Allocation | N/A |  |  |  |  |
| **INTERVENTIONS:** |  |  |  |  |  |
|  | N/A |  |  |  |  |
| **ASSESSMENTS:** |  |  |  |  |  |
| List basaline variables | X |  |  |  |  |
| List outcomes variables | X |  |  |  |  |
| List other data variables | X |  |  |  |  |

## 3.6. Sample size

## 3.7. Recruitment

In 2023, around 325 heart transplants (HT) were performed in Spain Given a median of 10-15 HT per center and considering that eight centers that routinely perform invasive coronary physiology are participating, data will be analyzed when 100 patients have completed first year follow-up.

## 3.8. Allocation

N/A

## 3.9. Blinding (masking)

### 3.9.1. Blinding mechanism

### 3.9.2. Emergency unblinding

N/A

## 3.10. Data collection

### 3.10.1. Trial procedures and evaluations

Data will be included in an online database specifically designed for the study on platform REDCap (Research Electronic Data Capture).

### 3.10.2. Retention

Clinical conditions, laboratory findings and clinical events will be assessed at one month and one year. A number will be assigned to each patient; their identity will not be disclosed in any case. All shared information will be anonymized. The principal investigator at each center will be responsible for keeping the data anonymized.

## 3.11. Data management

A number will be assigned to each patient; their identity will not be disclosed in any case. All shared information will be anonymized. The principal investigator at each center will be responsible for keeping the data anonymized.

Data will be processed in accordance with the protection legislation in force (Spanish Personal Data Protection and Guarantee of Digital Rights Act 3/2018, and Regulation (EU) 2016/679).

## 3.12. Statistical methods

### 3.12.1. Outcomes

The characteristics of the study population will be summarized through standard descriptive statistics. Continuous variables will be expressed as mean (± standard deviation) or median [interquartile range (IQR)], as appropriate. Discrete variables will be presented as absolute numbers and percentages. For the comparison of means, the Student's t test for independent measures or the non-parametric Mann-Whitney U test (in the case of dichotomous qualitative variables) and the ANOVA or non-parametric Kruskal test will be used as a hypothesis test. Wallis (in the case of non-dichotomous qualitative variables). For the bivariate analysis of the qualitative variables, the Chi-square test or Fisher's exact test will be used. Events will be compared between groups by using Kaplan-Meier curves. Cox regression analysis will be performed to adjust for confounding factors and to evaluate independent predictors of clinical events. A two-tailed p-value of 0.05 will be considered statistically significant. All statistical analyses will be performed by SPSS software version 20.

In 2023, around 325 heart transplants were performed in Spain (3). Given a median of 10-15 HT per center and considering that eight centers that routinely perform invasive coronary physiology are participating, data will be analyzed when 100 patients have completed first year follow-up.

### 3.12.2. Additional analyses

N/A

### 3.12.3. Analysis population and missing data

N/A

## 3.13. Data monitoring

## 3.14. Safety/harms

N/A

## 3.15. Auditing

N/A

# 4. ETHICS AND DISSEMINATION

## 4.1. Research ethics approval

This study adhered to the principles outlined in the Declaration of Helsinki. The approval was granted by the Ethics Committee for Investigation of Aragon (CEICA).

## 4.2. Protocol amendments

Protocol changes or modifications to this observational study will be reported in ClinicalTrials.gov.

## 4.3. Informed consent process

All the patients will sign the informed consent specifically designed for this study.

## 4.4. Confidentiality

Sociodemographic, clinical, laboratory and follow-up data of each patient will be included in a database specifically designed for the study. All variables will be included in the online data collection platform Redcap (Research Electronic Data Capture).  Each patient will be assigned a number; their identity will not be revealed in any case. All shared information will be anonymized.

## 4.5. Declaration of interests

None of the principal investigators have relevant relationships to the contents of this study to disclose.

## 4.6. Access to data

Only principal investigators will have access to the trial dataset. No disclosure of contractual agreements limit such access for investigators.

## 4.7. Dissemination policy

### 4.7.1. Trial results

Trial results will be reported via publication.

### 4.7.2. Authorship

Authorship eligibility guidelines will be established  according to the number of patients included by each center.

### 4.7.3. Reproducible research

N/A

# 5. STUDY ADMINISTRATION

## 5.1. Key contacts

**Central contact**

Georgina Fuertes Ferre

Interventional Cardiology Department Miguel Servet University Hospital. Zaragoza, Spain

0034 646 323 423

georginaff@hotmail.com

**Sponsor**

Goergina Fuertes Ferre

Interventional Cardiology Department Miguel Servet University Hospital. Zaragoza, Spain

Zaragoza, Zaragoza, 50009

Spain

0034 646 323 423

georginaff@hotmail.com

## 5.2. Funders

No funding.

## 5.3. Roles and responsibilities

### 5.3.1. Protocol contributors

**Index of microvascular resistance (IMR)-guided management of heart transplantation**

**IMR-Heart Transplant Study**

**PROTOCOL**

**SUMMARY**

| **Study title** | INDEX OF MICROVASCULAR RESISTANCE-GUIDED MANAGEMENT OF HEART TRANSPLANTATION |
| --- | --- |
| **Short Running Title** | IMR-HEART TRANSPLANT STUDY |
| **Investigators** | Georgina Fuertes Ferre  Ainhoa Pérez Guerrero  Teresa Blasco Peiró  Salvatore Brugaletta |
| **Study aim** | Follow-up assessment of heart transplant patients based on IMR |
| **Hypothesis** | The index of microvascular resistance will serve to reduce the number of endomyocardial biopsies in transplant patients. |
| **Study design** | Multi-center, prospective, observational |
| **Inclusion criteria** | Heart transplant patients >18 years. |
| **Exclusion criteria** | Patients presenting with hemodynamic instability after HT, including cardiogenic shock or severe coagulopathy. Patients with acute cellular rejection before intracoronary physiological assessment. Patients with bronchial asthma or bronchopathy with a positive bronchodilation test, which contraindicates the use of adenosine. Patients with epicardial coronary lesions with a resting physiological index ≤0.89 or ≤0.80 at hyperemia. Patients unlikely to cooperate in the study or with inability or unwillingness to give informed consent. |
| **Primary endpoint** | Number of biopsies performed in the first year.  IMR in transplant patients, in the first three months and one year. |
| **Secondary endpoints** | Cardiovascular mortality and IMR.  Cardiac allograft vasculopathy and IMR.  Acute cellular rejection and IMR.  IMR variation, in the first two months and at one year.  Combined endpoint of ACR, heart failure, re-transplantation, cardiovascular mortality and IMR.  Immunosuppressive therapy and IMR. |
| **Data analysis** | Observational, analytical |
| **Follow-up** | 1 year and annually for five years |

**STUDY VARIABLES**

| **Event** | **Screening** | **1-2 month(s)**  **post-transplantation** | **12 months**  **post-transplantation** | **Annual**  **for 5 years** |
| --- | --- | --- | --- | --- |
| **Criteria** **for**  **inclusion/exclusion** | X |  |  |  |
| **Informed**  **consent** | X |  |  |  |
| **Laboratory test** | X | X |  |  |
| **Echocardiogram** | X | X | X |  |
| **Medication** | X | X | X |  |
| **Functional invasive coronary test** |  | X | X |  |
| **Events during**  **follow-up** |  |  | X | X |

**MAIN CONTACTS**

| **Principal investigators** | **Georgina Fuertes Ferre**  Interventional Cardiology Unit  Miguel Servet University Hospital, Zaragoza  e-mail:georginaff@hotmail.com |
| --- | --- |
|  | **Ainhoa Pérez Guerrero**  Interventional Cardiology Unit  Miguel Servet University Hospital, Zaragoza  e-mail: ainhoaperezguerrero @gmail.com |
|  | **Teresa Blasco Peiró**  Heart Failure and Transplant Unit  Miguel Servet University Hospital, Zaragoza  e-mail: tblascop@gmail.com |
|  | **Salvatore Brugaletta**  Interventional Cardiology Unit  Clinic University Hospital, Barcelona  e-mail: sabrugaletta@gmail.com |
| **Study sponsor** | **Georgina Fuertes Ferre**  Interventional Cardiology Unit  Miguel Servet University Hospital, Zaragoza  e-mail:georginaff@hotmail.com |

**ABBREVIATIONS**

**AAR:** acute allograft rejection

**ACR:** acute cellular rejection

**CFR:** coronary flow reserve

**EMB:** endomyocardial biopsy

**FFR:** fractional flow reserve

**HT:** heart transplant

**IMR:** index of microvascular resistance

**IST:** immunosuppressive therapy

**MVD:** microvascular dysfunction

**Pa:** aortic pressure

**Pah:** aortic pressure at hyperemia

**Pd:** distal coronary pressure

**Pdh:** distal coronary pressure at hyperemia

**RFR:** resting full-cycle ratio

**Tmnh:** mean transit time at hyperemia

**Tmnr:** mean transit time at rest

**INTRODUCTION AND RATIONALE**

Heart transplant (HT) is considered the treatment of choice for patients with advanced heart failure refractory to medical treatment or devices.

Acute allograft rejection (AAR) is the main cause of mortality and re-transplantation,  particularly during the first year. The immune response is classified into acute cellular rejection (ACR) when mediated by T lymphocytes, and humoral rejection when involving B lymphocytes and antibody production. Of note, up to 20% of HT patients experience at least one episode of ACR during the first year post-transplantation (1). Advances in immunosuppressive therapy (IST), donor heart evaluation, surgical techniques and post-HT care have led to a reduction in ACR, improving survival over time (2-3). Post-transplant IST includes three basic components: a calcineurin inhibitor (currently preferred Tacrolimus), an antiproliferative agent (mycophenolate mofetil), and steroids. On the other hand, proliferation signal inhibitor (mTOR) drugs (everolimus and sirolimus) are primarily used for CAV (3-4). CAV is the main cause of mortality after the first year of transplantation. It is characterized by diffuse intimal thickening affecting both coronary epicardial and microcirculation (2,4).

The vast majority of ACR occur asymptomatically with normal ventricular function, and thus being detected by endomyocardial biopsy (EMB). EBM is the "gold standard" to guide post-HT treatment (4). The graduation of the RAC is detailed in the 2005 review of the ISHLT (5). Due to intra- and inter-observer variabilities in determining different degrees of slight-moderate rejection, an update was published in the 2010 document. In the International Society of Heart and Lung Transplantation (ISHLT) guidelines, EMB was made a IIaC recommendation for the detection of rejection (2). EMB is repeatedly performed during the first year after HT and is associated with complications that, despite infrequent, can be potentially serious, such is the case of cardiac perforation. Moreover, EMB diagnostic yield is wide, with a high variability between observers and a non-negligible rate of false positives and negatives (6).

In order to avoid the inconveniences of EMB, non-invasive techniques have been studied to detect rejection, with some positive results. However, none of these techniques has been able to replace EMB (7).

Various studies have demonstrated the association between ACR, microvascular dysfunction (MVD) and cardiac allograft vasculopathy (CAV) (8). IMR is a quantitative and specific index for coronary microcirculation (9-10).  An increased IMR in the graft has been associated with higher all-cause mortality and adverse cardiac events regardless of epicardial vasculopathy (11). Several IMR cut-off (from 15 to >25) have been associated with ACR within the first year after HT. Patients in whom IMR decreases or does not change one year after HT have a higher event-free rate than those patients in whom the IMR increases (12-13). However, no study has evaluated IMR impact on post-HT management. In presence of a low IMR value, EMB could be performed less frequently; on the contrary, if the IMR value is high, immunosuppression therapy could be modified by an earlier administration of mTor-inhibiting drugs or prescribing calcium antagonists, which are known to improve microvascular function.

Our aim will be to assess if post-HT patient management may be safely modified based on IMR value.

**STUDY DESIGN**

**ENDPOINTS**

*The main objective* of the study is to evaluate IMR values and number of EMB performed in the first year after HT.

S*econdary endpoints* are:

- Analyze the relationship between ACR and IMR.
- Analyze the relationship between CV mortality and IMR.
- Analyze the relationship between CAV and IMR.
- Analyze the relationship between the variation IMR value in the first two months and one year, and cardiovascular events.
- Analyze the relationship between the combined endpoint of ACR, heart failure, re-transplantation, CV mortality and IMR.
- Assessment of immunosuppressive therapy after IMR results.

All endpoints will be evaluated at 1 year, and annually thereafter for up to 5 years.

**HYPOTHESIS**

Number of EMB performed in the first year post-HT could vary depending on IMR values. Low IMR values in the first three months could reduce the number of protocol EMB. Meanwhile, high IMR values would not modify the number of EMB performed, and could even contribute to intensify immunosuppressive therapy.

**METHODS**

This is a multi-center, prospective, observational study of HT patients at the participating centers. The recruitment period of the study is two years.

*Informed consent:* All the patients will sign informed consent specifically designed for this study.

***Inclusion criteria***

- Heart transplant patients >18 years.
- Patients who have received and signed informed consent.

***Exclusion criteria***

- Patients with hemodynamic instability after HT, including cardiogenic shock or severe coagulopathy.
- Patients with acute cellular rejection before intracoronary physiological assessment.
- Patients with bronchial asthma or bronchopathy with a positive bronchodilation test, which contraindicates the use of adenosine.
- Patients with epicardial coronary lesions with a resting physiological index ≤0.89 or ≤0.80 at hyperemia.
- Patients unlikely to cooperate or with inability or unwillingness to give informed consent.

***Procedure***

**Invasive physiological evaluation of the coronary microcirculation**

Patients included will undergo a coronary angiography between the first and third month after HT.  This coronary angiography is part of the follow-up protocol in most of the centers. Assessment of IMR, coronary flow reserve (CFR) and fractional flow reserve (FFR) will be performed using the standard technique (9-10,14).

The left anterior descending coronary artery will be evaluated in all patients. Circumflex or right coronary artery could be additionally evaluated at operator’s discretion. An intracoronary pressure and temperature sensor-tipped guidewire (Pressure Wire TM X guide- wire 0.014’, Abbott, IL, USA) will be used to perform the measurements. The tip pressure sensor will be advanced into the mid-to-distal portion of the evaluated vessel. Baseline aortic pressure (Pa) and distal intracoronary pressure (Pd) will be obtained to calculate the resting index Pd/Pa. To measure the mean transit time (Tmn) under basal conditions, intracoronary administration of 3 mL of room-temperature saline will be manually injected three times in succession (3 mL/s). Then maximal hyperemia will be induced using adenosine iv (140 to 180 mg/kg/min) and three additional intracoronary room temperature saline boluses of 3 ml will be administered to determine the mean transit time at hyperemia (Tmnh). Finally, fractional flow reserve (FFR), coronary flow reserve (CFR) and IMR will be calculated using the software Coroventis Coroflow (*Coroventis Abbott, Uppsala, Sweden)*.

- FFR is defined as the ratio of maximal coronary blood flow in a diseased artery to maximal coronary blood flow in the same artery without stenosis. FFR is a surrogate marker of inducible myocardial ischemia caused by epicardial coronary stenosis.

- CFR is the ratio of hyperemic to baseline flow and is a marker of the integrity of both epicardial and microvascular coronary circulation. Therefore, CFR represents the microvascular status when there is no significant epicardial disease.

- IMR is the minimum achievable coronary microcirculatory resistance and a more specific marker of the coronary microcirculation. It is calculated as the ratio of distal coronary pressure to coronary flow at hyperemia and presented in units (mmHg.s).

The physiological study assessing IMR, CFR and FFR will be repeated again one year after HT.

***Biopsies and immunosuppressive therapy***

Based on IMR results:

- If the IMR is less than 15, the number of biopsies could be reduced or kept the same. No immunosuppressive therapy changes would be made.

- If the IMR is 15 or greater, immunosuppressive therapy could be intensified or maintained the same. Number of biopsies would be performed as usual per protocol.

Given the observational characteristics of the study, decisions regarding clinical patient management will depend on physician discretion considering patient clinical condition and other complementary tests.

***Data and follow-up***

Clinical conditions, laboratory findings and clinical events will be assessed at one month and one year. Data will be included in an online database specifically designed for the study on platform REDCap (Research Electronic Data Capture).

A number will be assigned to each patient; their identity will not be disclosed in any case. All shared information will be anonymized. The principal investigator at each center will be responsible for keeping the data anonymized.

***Definition of principal variables***

- **Pa:** basal proximal (aortic) pressure.
- **Pah:** aortic pressure at maximal hyperemia.
- **Pd:** basal distal pressure.
- **Pdh:** distal pressure at maximal hyperemia.
- **Tmnr:** Mean transit time of the saline injected at rest.
- **Tmnh:** Mean transit time of the saline injected at maximal hyperemia.
- **RFR:** Resting index calculated on the basis of the lowest Pd/Pa value throughout the cardiac cycle. A value ≤0.89 is associated with functionally significant coronary stenosis.
- **FFR:** Hyperemic index calculated by Pdh/Pah quotient. FFR ≤0.80 is associated with functionally significant coronary stenosis.
- **CFR (coronary flow reserve):** This parameter explores the maximal vasodilation capacity of the entire coronary bed (epicardial and microvascular), and hence the increase in coronary flow compared with the resting value.

The fundamental principle is the inversely proportional relationship between the coronary flow and transit time (TT) of a bolus of intracoronary saline, estimated on the basis of the change in temperature. CFR is considered to be low when is <2; normal >2.5.

CFR= 1/(Tmnh/Tmnr)

- **IMR (index of microcirculatory resistance):** This index reflects microvascular function regardless of epicardial circulation. It is obtained in a state of maximal hyperemia. A value ≥25 is considered pathological; normal <20. In our study, an IMR ≥15 will be considered high (8).

IMR= Pdh x Tmnh.

***Definition of events***

- **Acute cellular rejection (ACR**): A ≥2R degree according to the 2010 ISHLT system. Two or more focal infiltration points associated with myocyte injury in EMB. Diffuse infiltration with multi-focal myocyte injury with/without oedema, hemorrhage or vasculitis (2).
- **Cardiovascular mortality:** Caused by cardiovascular disease or unknown death.
- **Cardiac allograft vasculopathy:** Accelerated fibroproliferative process characterized by diffuse, concentric and longitudinal thickening of the intima of the vascular tree of the graft, affecting everything from the major epicardial arteries to the coronary microvasculature (2).
- **Combined endpoint:** ACR, heart failure, re-transplantation and cardiovascular mortality.
- **Cardiac catheterization complications:** any complication (major or minor) related to the performance of EMB, coronary angiography and functional study.

***Changes in EMB protocol based on coronary microvascular resistance***

- Each center will specify the number of annual biopsies performed based on IMR values obtained between the first two months after HT.

***Statistical analysis***

The characteristics of the study population will be summarized through standard descriptive statistics. Continuous variables will be expressed as mean (± standard deviation) or median [interquartile range (IQR)], as appropriate. Discrete variables will be presented as absolute numbers and percentages. For the comparison of means, the Student's t test for independent measures or the non-parametric Mann-Whitney U test (in the case of dichotomous qualitative variables) and the ANOVA or non-parametric Kruskal test will be used as a hypothesis test. Wallis (in the case of non-dichotomous qualitative variables). For the bivariate analysis of the qualitative variables, the Chi-square test or Fisher's exact test will be used. Events will be compared between groups by using Kaplan-Meier curves. Cox regression analysis will be performed to adjust for confounding factors and to evaluate independent predictors of clinical events. A two-tailed p-value of 0.05 will be considered statistically significant. All statistical analyses will be performed by SPSS software.

***Confidentiality and data protection***

Data will be processed in accordance with the protection legislation in force (Spanish Personal Data Protection and Guarantee of Digital Rights Act 3/2018, and Regulation (EU) 2016/679).

***Ethical considerations***

The study will be conducted according to the protocol, in agreement with the ethical principles of the Declaration of Helsinki. It will not begin until authorization has been obtained from the Ethics Committee.

**BIBLIOGRAPHY**

1. L.H. Lund, L.B. Edwards, A.Y. Kucheryavaya, C., et al., The registry of the International Society for Heart and Lung Transplantation: thirty-second official adult heart transplantation report- 2015; focus theme: early graft failure, J. Heart Lung Transplant. 34 (2015) 1244–1254.
2. The International Society of Heart and Lung Transplantation Guidelines for the care of heart transplantation recipients. Task Force 2: Immunosuppression and Rejection. J Heart Lung Transplant. 2010.;29:914-956.
3. Khush KK, Cherikh WS, Chambers DC, et al. The International Thoracic Organ Transplant Registry of the International Society for Heart and Lung Transplantation: Thirty-Sixth Adult Heart Transplantation Report—2019; focus theme: donor and recipient size match. J Heart Lung Transplant. 2019;38:1056–1066.
4. Marboe CC, Billingham M, Eisen H et al. Nodular endocardial infiltrates (Quilty lesions) cause significant variability in diagnosis of ISHLT 2 Grade 2 and 3A rejection in cardiac allograft recipients. J Heart Lung Transplant. 2005;24: S219-S226.
5. Stewart S, Winters GL, Fishbein MC, et al. Revision of the 1990 working formulation for the standardization of nomenclature in the diagnosis of heart rejection. J Heart Lung Transplant. 2005;24:1710–1720.
6. Saraiva F, Matos V, Gonçalves L, Antunes M, Providência LA. Complications of endomyocardial biopsy in heart transplant patients: a retrospective study of 2117 consecutive procedures. Transplant Proc. 2011;43:1908–1912.
7. Khachatoorian Y, Khachadourian V, Chang E et al. Noninvasive biomarkers for prediction and diagnosis of heart transplantation rejection. Transplant Rev (Orlando). 2021;35:1005-90.
8. Harmann NE, Wellnhofer E, Knosalia C et al. Prognostic impact of microvasculopathy on survival after heart transplantation: evidence from 9713 endomyocardial biopsies. Circulation. 2007;116:1274-1282.
9. Fearon WF, Balsam LB, Farouque HMO, et al. Novel index for invasively assessing the coronary microcirculation. Circulation. 2003;25:3129-32.
10. Fearon WF, Kobayashi Y. Invasive assessment of the coronary microvasculature: the index of microcirculatory resistance. Circ Cardiovasc Interv. 2017;10.
11. Ahn JM, Zimmermann FM, Gullestad L, et al. Microcirculatory resistance predicts allograft rejection and cardiac events after heart transplantation. J Am Coll Cardiol. 2021;78:2425–2435. 10- Yang HM, Khush K, Luikart H, et al. Invasive assessment of coronary physiology predicts late mortality after heart transplantation. Circulation. 2016;133:1945–1950.
12. Okada K, Honda Y, Luikart H, et al. Early invasive assessment of the coronary microcirculation predicts subsequent acute rejection after heart transplantation. Int J Cardiol. 2019;290:27–32. 12- DEC GW, Narula J Probing the Microvasculature for Long-Term Gains in Heart Transplant Recipients. J Am Coll Cardiol. 2021;78:2436-2438.
13. Lee JM , Mo KH, Cho JO et al. Coronary Microcirculatory Dysfunction and Acute Cellular Rejection After Heart Transplantation Circulation. 2021;144:1459–1472.
14. Kunadian V, Chieffo A, Camici PG, Berry C, Escaned J, Maas AHEM, et al. An EAPCI Expert Consensus Document on Ischaemia with Non-Obstructive Coronary Arteries in Collaboration with European Society of Cardiology Working Group on Coronary Pathophysiology & Microcirculation Endorsed by Coronary Vasomotor Disorders International Study Group. EuroIntervention. 2021 Jan; 16(13).

### 5.3.2. Sponsor and funder

- Georgina Fuertes Ferre: This author has participated in the study design; collection, management, analysis, and interpretation of data; writing of the report; and the decision to submit the report for publication.

- Ainhoa Perez Guerrero: This author has participated in the study design; collection, management, analysis, and interpretation of data; writing of the report; and the decision to submit the report for publication.

- Teresa Blasco Peiro: This author has participated in the study design and  the manuscript review.

- Salvatore Brugaletta: This author has participated in the study design and  themanuscript review.

### 5.3.3. Trial committees

- Coordinating Centre: Miguel Servet University Hospital.

- Data management team: Online database *REDCap, from the Spanish Society* of *Cardiology*,

# 6. APPENDICES

## 6.1. Informed consent materials

**DOCUMENTO DE INFORMACIÓN PARA EL PARTICIPANTE**

**Título de la investigación:** **“IMR-Heart transplant study”**

**Promotor**: Georgina Fuertes Ferre. Ainhoa Pérez Guerrero.

**Investigador Principal:** Georgina Fuertes Ferre **Tfno:** 976765500- Ext 5160 **mail:**

**Centro** Hospital Universitario Miguel Servet, Zaragoza.

1. **Introducción:**

Nos dirigimos a usted para solicitar su participación en un proyecto de investigación que estamos realizando en ____________. Su participación es absolutamente voluntaria, en ningún caso debe sentirse obligado a participar, pero es importante para obtener el conocimiento que necesitamos. Este proyecto ha sido aprobado por el Comité de Ética. Antes de tomar una decisión es necesario que:

- lea este documento entero

- entienda la información que contiene el documento

- haga todas las preguntas que considere necesarias

- tome una decisión meditada

- firme el consentimiento informado, si finalmente desea participar.

Si decide participar se le entregará una copia de esta hoja y del documento de consentimiento firmado. Por favor, consérvelo por si lo necesitara en un futuro.

1. **¿Por qué se le pide participar?**

Se le solicita su colaboración porque usted es un paciente trasplantado cardiaco, y es en este grupo de pacientes donde se lleva a cabo este estudio.

En el estudio participarán varios centros españoles, y se realizará también en pacientes trasplantados.

1. **¿Cuál es el objeto de este estudio?**

La biopsia endomiocárdica es la técnica más empleada para guiar el manejo y tratamiento post-trasplante cardiaco. Es la prueba que actualmente mejor predice el riesgo de rechazo. Si bien se realiza de forma rutinaria, es una prueba invasiva cuyo uso no está exento de complicaciones. Se están estudiando algunas técnicas que podrían reducir el número de biopsias que se realizan durante el seguimiento del paciente trasplantado. Existe un índice que mide la microcirculación coronaria que se llama índice de resistencia microvascular (IMR), y este índice se ha asociado al rechazo agudo celular en el trasplante cardiaco. El IMR se mide durante la coronariografía que se realiza habitualmente de forma asistencial en el seguimiento. **El objetivo principal de este estudio es analizar cómo puede ayudar la determinación del IMR en el seguimiento de los pacientes trasplantados.** En función de los resultados del IMR, se podría reducir el número de biopsias o se podría intensificar el tratamiento inmunosupresor.

1. **¿Qué tengo que hacer si decido participar?**

Si accede a participar en el estudio, la fecha de realización de dos de los cateterismos que forman parte de su seguimiento podría variar un poco, pero se intentará que no.

Durante el cateterismo se llevará a cabo una valoración fisiológica de la función coronaria para la obtención del IMR. La valoración fisiológica es un proceso asistencial de la práctica habitual cuyo consentimiento firmará de forma específica.

Tras los resultados del IMR y del resto de pruebas complementarias que le hayan realizado, sus médicos responsables decidirán si pueden reducir número de biopsias futuras, intensificar el tratamiento inmunosupresor o bien mantener el mismo protocolo que se viene realizando previamente en su hospital.

Durante 5 años, el investigador de su centro seguirá su evolución a través de su historia clínica. Usted mantendrá las revisiones rutinarias por sus médicos especialistas, no tendrá que acudir a ninguna visita extra ni se precisará la realización de más pruebas complementarias.

1. **¿Qué riesgos o molestias supone?**

El cateterismo cardiaco y estudio de microcirculación se trata de un procedimiento asistencial de la práctica habitual. Los riesgos y complicaciones de estas técnicas serán explicadas por su médico y firmará el consentimiento específico.

Las molestias de este estudio son aceptar la cesión de sus datos para fines de investigación.

1. **¿Obtendré algún beneficio por mi participación?**

Al tratarse de un estudio de investigación orientado a generar conocimiento usted no obtendrá ningún beneficio por su participación si bien contribuirá al avance científico y al beneficio social.

Usted no recibirá ninguna compensación económica por su participación.

1. **¿Cómo se van a tratar mis datos personales?**

Los datos recogidos para el estudio estarán identificados por un número, no se incluirá ninguna información que pueda identificarle. Solo sus médicos e investigador/es del estudio de su centro podrán relacionar sus datos. Su participación en el estudio quedará reflejada en su historial médico.

***Información básica sobre protección de datos:***

*Responsable del tratamiento:* …………………………………

*Finalidad:* Sus datos personales serán tratados exclusivamente para el trabajo de investigación a los que hace referencia este documento.

*Legitimación:* El tratamiento de los datos de este estudio queda legitimado por su consentimiento a participar.

*Destinatarios*: No se cederán datos a terceros salvo obligación legal.

*Derechos*: Podrá ejercer sus derechos de acceso, rectificación, supresión y portabilidad de sus datos, de limitación y oposición a su tratamiento, de conformidad con lo dispuesto en la LO 3/2018 de Protección de Datos Personales y garantía de los derechos digitales y el Reglamento General de Protección de Datos (RGPD 2016/679) ante el delegado de protección de datos de la institución, dirigiendo un correo electrónico a la dirección dpd@salud.aragon.es en caso de realizarse en Aragón o a ……………………….............en caso de realizarse en otra Comunidad …………………………………

Podrá consultar información adicional y detallada en el Registro de Actividades de Tratamiento del Gobierno de Aragón, en el siguiente enlace: [https://aplicaciones.aragon.es/notif_lopd_pub/details.action?fileId=731](https://www.spiritprotocol.com/api/docx/https://aplicaciones.aragon.es/notif_lopd_pub/details.action?fileId=731); o en el siguiente enlace si se realiza en otra Comunidad …………………………………………….

Así mismo, en cumplimiento de los dispuesto en el RGPD, se informa que, si así lo desea, podrá acudir a la Agencia de Protección de Datos (https://www.aepd.es) para presentar una reclamación cuando considere que no se hayan atendido debidamente sus derechos.

El tratamiento de sus datos personales se realizará utilizando técnicas para mantener su anonimato mediante el uso de códigos aleatorios, con el fin de que su identidad personal quede completamente oculta durante el proceso de investigación.

A partir de los resultados del trabajo de investigación, se podrán elaborar comunicaciones científicas para ser presentadas en congresos o revistas científicas, pero se harán siempre con datos agrupados y nunca se divulgará nada que le pueda identificar.

1. **¿Cómo se van a tratar mis muestras biológicas?**

**NO** se recogen muestras biológicas en este estudio.

1. **¿Quién financia el estudio?**

Este estudio no está financiado.

1. **¿Se me informará de los resultados del estudio?**

Usted tiene derecho a conocer los resultados del presente estudio, tanto los resultados generales como los derivados de sus datos específicos. También tiene derecho a no conocer dichos resultados si así lo desea. Por este motivo en el documento de consentimiento informado le preguntaremos qué opción prefiere. En caso de que desee conocer los resultados, el investigador le hará llegar los resultados.

En ocasiones al realizar un proyecto de investigación se encuentran hallazgos inesperados que pueden ser relevantes para la salud del participante. En el caso de que esto ocurra nos pondremos en contacto con usted para que pueda acudir a su médico habitual.

1. **¿Puedo cambiar de opinión?**

Su participación es totalmente voluntaria, puede decidir no participar o retirarse del estudio en cualquier momento sin tener que dar explicaciones y sin que esto repercuta en su atención sanitaria. Basta con que le manifieste su intención al investigador principal del estudio. En caso de que decida retirarse del estudio puede solicitar la destrucción de los datos, muestras u otra información recogida sobre usted.

1. **¿Qué pasa si me surge alguna duda durante mi participación?**

En la primera página de este documento está recogido el nombre y el teléfono de contacto del investigador responsable del estudio. Puede dirigirse a él en caso de que le surja cualquier duda sobre su participación.

Muchas gracias por su atención, si finalmente desea participar le rogamos que firme el documento de consentimiento que se adjunta y le reiteramos nuestro agradecimiento por contribuir a generar conocimiento científico.

**DOCUMENTO DE CONSENTIMIENTO INFORMADO**

**Título del PROYECTO: “IMR-Heart transplant study”**

  Yo, .............................................................................. (nombre y apellidos del participante)

He leído la hoja de información que se me ha entregado.

He podido hacer preguntas sobre el estudio y he recibido suficiente información sobre el mismo.

He hablado con: ...........................................................................(nombre del investigador)

Comprendo que mi participación es voluntaria.

Comprendo que puedo retirarme del estudio:

1) cuando quiera

2) sin tener que dar explicaciones

3) sin que esto repercuta en mis cuidados médicos/mi relación con el investigador.

Presto libremente mi consentimiento para participar en este estudio y doy mi consentimiento para el acceso y utilización de mis datos conforme se estipula en la hoja de información que se me ha entregado.

Deseo ser informado sobre los resultados del estudio: sí     no   (marque lo que proceda)

Doy mi conformidad para que mis datos clínicos sean revisados por personal ajeno al centro, para los fines del estudio, y soy consciente de que este consentimiento es revocable.

He recibido una copia firmada de este Consentimiento Informado.

| Firma del participante: |  |
| --- | --- |
| Fecha: |  |
|  |  |

He explicado la naturaleza y el propósito del estudio al paciente mencionado

| Firma del Investigador: |  |
| --- | --- |
| Fecha: |  |
