# Supplementary material for: Index microvascular resistance (IMR) in heart transplant patients (IMR-HT study): Study protocol
Source: PLoS One. 2025 May 16;20(5):e0315053. doi: 10.1371/journal.pone.0315053 (PMC12084034; doi:10.1371/journal.pone.0315053)
Supplement: S2 Table — (DOCX) [file pone.0315053.s002.docx]

| Centers | Investigators |
| --- | --- |
| Miguel Servet University Hospital | Georgina Fuertes Ferré, Teresa Blasco Peiró, Ainhoa Pérez Guerrero |
| La Fe University Hospital | Jean Paul Vilchez Tschischke, Luis Almenar Bonet, Raquel López Vilella, Jose Luis Diez Gil |
| Bellvitge Hospital | Josep Gómez-Lara, José González Costello |
| Asturias Central University Hospital | Paula Antuña Álvarez, Vanesa Alonso Fernández |
| 12 Octubre University Hospital | Fernando Sarnago Cebada, Dolores García Cosio |
| Reina Sofía de Córdoba University Hospital | Francisco Hidalgo Lesmes, Amador López Granados |
| Virgen de la Arrixaca University Hospital | Ramón López Palop, Iris Paula Garrido |
| Virgen del Rocío University Hospital | Rosa María Cardenal Piris, Diego Rangel Sousa |

**Supplementary Table 1. Site centers and investigators**
