# Supplementary material for: Index microvascular resistance (IMR) in heart transplant patients (IMR-HT study): Study protocol
Source: PLoS One. 2025 May 16;20(5):e0315053. doi: 10.1371/journal.pone.0315053 (PMC12084034; doi:10.1371/journal.pone.0315053)
Supplement: Attachment — Submitted filename: Rebuttal letter 18.02.docx [file pone.0315053.s004.docx]

18 th February 2025

Dear PLOS One Editor and Reviewers,

We sincerely thank you for the detailed and constructive feedback provided on our manuscript titled “Index microvascular resistance (IMR)-guided management of heart transplantation (IMR-HT study): Study Protocol.” We have carefully addressed all the comments and made the necessary revisions to improve the quality of our work. Below, we provide a point-by-point response to the reviewers’ comments, highlighting the changes made in the revised manuscript.

For clarity, the editor and reviewers’ comments are presented in bold, followed by our responses in regular text. Revised text is highlighted in the manuscript for easy reference.

**Response to Editors' comments:**

**E: While the study rationale is well-presented, several issues need clarification. The risks associated with using saline injections and adenosine for IMR assessment are not discussed, and there is no comparison of IMR with other noninvasive diagnostic tools.**

R: We thank the Editor for this comment. In our study, IMR will be evaluated by the standard bolus thermodilution technique. Hyperemia is commonly achieved with the use of intravenous adenosine, which can cause adverse symptoms *(Mejia-Renteria H, J Am Heart Assoc. 2024)*. Particularly in heart transplant patients, Duran SR et al. (*Pediatr Radiol. 2021)* showed no complications when using adenosine at a rate of 140 mcg/kg/min in 16 pediatric patients undergoing MR stress perfusion cardiac magnetic resonance. The risks associated with using saline injection and adenosine for IMR assessment have been added to the discussion, page 17:

*“IMR is a quantitative and specific index of coronary microcirculation that requires the use of a coronary guide and hyperemia to be analyzed. Both adenosine and the use of a coronary wire could cause adverse symptoms (20). However, the rate of potential serious adverse events is equivalent to that of routine diagnostic coronary angiography (<1%) (21). Particularly in heart transplant patients, Duran SR et al. showed no complications when using adenosine at a rate of 140 mcg/kg/min in 16 pediatric patients undergoing MR stress perfusion cardiac magnetic resonance”*

R: We agree with the Editor, there is no comparison of IMR with other noninvasive diagnostic tools. The modern genetic techniques have not been implemented in routine clinical practice in most of our Spanish centers. Probably, in the near future, both diagnostic tools may be useful for HT patients. We have added this limitation to the discussion page 17:

*“There are no studies comparing IMR with other non-invasive tools for the detection of acute allograft rejection. However, rather than a substitute, IMR could be a complementary technique to gene expression profiling in HT patients”.*

**E: The hypotheses are framed using non-refutable language, and clearer, more testable formulations are suggested to strengthen the study’s objectives.**

R: We thank the Editor for his/her valuable comment. More clearer and testable formulations have been framed: hypothesis, page 6:

***“****There are few studies that evaluate IMR after a heart transplant. High IMR baseline value will identify patients at higher risk and will lead to modifications in the number of biopsies and/or immunosuppressive therapy within one year. Patients with an increase in IMR over the first year will have a higher rate of cardiac events”.*

**E: The methodology for assigning patients to comparison groups lacks clarity, particularly regarding the configuration of groups based on IMR values and changes in EMB or immunosuppressive therapy. It is recommended to compare outcomes where IMR influenced treatment decisions or to analyze data against historical cases from the same centers.**

R: Our aim when proposing a different management strategy based on IMR baseline values (<15 or ≥15) is not to compare the groups. Our goal is to observe the number of biopsies that clinicians can avoid when the IMR baseline value is low, as well as possible changes in immunosuppressive therapy when the IMR baseline value is high. We were hoping to reduce the number of biopsies that we, as interventional cardiologists, routinely perform while managing potential serious complications. However, the preliminary analysis of the first fifty patients has shown that only one center reduced the number of biopsies following the evaluation of a low IMR value during the first month after HT. Clinicians are not yet fully “comfortable” with decreasing the number of biopsies based solely on IMR. In this observational trial, only IMR measurement was a requisite to participate, modification of HT management was not mandatory. After discussing internally we have decided to change the primary objective and move the reduction in the number of biopsies within the first year to a secondary objective. The study methodology remains unchanged. These modifications have been also updated in clinicaltrials.gov.

The study's primary objective and secondary end points have been clarified: page 4, lines 13-14; page 6, lines 1-3; page 13, lines 1-3 and 14-15:

*“Our aim is to assess IMR values in heart transplant patients during the first months and at one year. Secondary endpoints include evaluating changes in management (number of EMBs, modifications in immunosuppressive therapy) after knowing baseline IMR values, as well as evaluating the prognostic implications of IMR measured in the first months and at one year”.*

**E: Additionally, discrepancies between the main file and the protocol file, including missing sections, inconsistent references, and grammatical errors, need to be resolved.**

R: We have resolved discrepancies between the protocol and the manuscript and enhanced the quality of the English throughout both texts.

**E: The definition of IMR based on mean transit time should be consistently presented across documents, and the study’s data sharing plan should specify where data will be available after publication.**

R: The definition of IMR has been standardized across all documents following the review: page 11, lines 4-6.

*“IMR is defined as the distal coronary pressure divided by the inverse of the hyperemic mean transit time and is expressed in units of mmHg.s”*.

R: The study’s data sharing plan will be available in clinicaltrials.gov after publication.

**Response to Reviewer 1 comments:**

**R1: Abstract; the correlation between number of biopsies and IMR is not clear**

R: Thank you for your suggestion. The correlation between the number of biopsies and IMR has been clarified in the abstract, page 4:

***“****The index of microvascular resistance (IMR) is a specific physiological parameter used to assess microvascular function. Invasive coronary assessment has been shown to be both feasible and safe. Detection of coronary microvascular dysfunction (MCD) by IMR may help to identify high risk HT patients. In fact, an increased IMR measured early after HT has been associated with AAR, higher all-cause mortality and adverse cardiac events. A high IMR value early after HT may identify patients at higher risk who require increased surveillance or adjustments in immunosuppressive therapy. Conversely, a low IMR value may support reducing the number of EMBs. Our aim is to evaluate IMR in heart transplant patients within the first year. Changes in management after knowing IMR values and prognostic implications of IMR in a long term follow up will also be assessed”.*

**R1: Methods: adenosine is quite used, do authors have experience about regadenoson?**

R: We strongly agree, adenosine is the most used agent to induce hyperemia. Specifically in heart transplant patients, all published data about assessing IMR in HT patients use adenosine to induce hyperemia (Lee JM, et al; Okada K, et al; Ahn J-M, et al.; Yang HM, et al.; Dec GW, et al). ^8-10, 22-23^ . There is little data about the use of regadenoson in heart transplant patients. Lazarus JJ et al (J Nucl Cardiol. 2020) reported no serious complications when using regadenoson in 123 HT patients undergoing positron emission tomography stress testing. Most of the authors have some experience with regadenoson; however, it has a longer half-life and is not available in many centers in our country.

**R1: Methods: sample size calculation is needed**

R: We thank the reviewer for this comment. Our study is observational, with the primary endpoint assessing IMR in HT patients. For this reason, we believe no sample size calculation is needed. This has been clarified in the manuscript on page 15, lines 14-18:

*“Given the exploratory nature of the study, no formal power calculation is required. In 2023, approximately 325 heart transplants were performed in Spain. With a median of 10–15 HTs per center and considering the participation of eight centers that routinely perform invasive coronary physiology, data will be analyzed once 100 patients have completed their first-year follow-up”.*

**R1: Discussion: personally, I find this proposal very interesting. My concern is: are authors sure that performing 2 microcircle evaluation per year is safer/ more cost effective than performing EBM? and how many EBMs do they think will be spared?**

R: According to the literature, the complication rate associated with EMB ranges from 0.71% to 9.2%, with cardiac perforation being one of them (*Saraiva F, et al., Transplant Proc. 2011*). On the other hand, complications related to coronary functional studies occur in less than 1% of cases (*Keulards DCJ, et al., EuroIntervention. 2021).*

These modifications are detailed in discussion, page 17:

*“IMR is a quantitative and specific index of coronary microcirculation that requires the use of a coronary guide and hyperemia to be analyzed. Both adenosine and the use of a coronary wire could cause adverse symptoms. However, the rate of potential serious adverse events is equivalent to that of routine diagnostic coronary angiography (<1%). Particularly in heart transplant patients, Duran SR et al. showed no complications when using adenosine at a rate of 140 mcg/kg/min in 16 pediatric patients undergoing MR stress perfusion cardiac magnetic resonance”*

R: We initially thought that at least one EMB per patient and center would have been spared. However, as detailed above, in total only one EMB was spared in one center (our promoter center). Unfortunately, we have observed our clinicians are not fully convinced in reducing the number of biopsies based on IMR results. However, we believe this observational study will serve as a first step in introducing microvascular function evaluation in our heart transplant patients. Our intention is to conduct a randomized trial that spares EMB based on IMR results. This has also been added to the text, page 12, lines 11-12.

*“The results of this observational trial will help in the conduct of a randomized trial in which an EMB will be spared based on the IMR value”.*

**Response to Reviewer 2 comments:**

**R2: Treatments: It is clearly stated that all HT patients must run through invasive coronary assessment as part of their follow up treatment, which currently includes repetitive EMB. The risks of performing repetitive EMB are discussed, emphasizing the notion that reducing EMB would be a favorable outcome for patients. However, a sound analysis pondering the risks (if any) of performing repetitive room temperature saline injections and adenosine infusion to cause maximal hyperemia in HT patients are not discussed, further, a pondered discussion of pro/con features of IMR versus other noninvasive diagnostic tools is missing.**

R: We agree with your observation and have revised the text accordingly. The updated section is located on page 17, lines 3-15.:

*“In recent years, advanced methods like gene expression profiling and plasma donor-derived cell-free DNA have also been introduced. However, their results have been highly variable, and these techniques have not been implemented in routine clinical practice in most of our centers (18,19). For these reasons, none of these techniques has still been able to replace EMB. IMR is a quantitative and specific index of coronary microcirculation that requires the use of a coronary guide and hyperemia to be analyzed. Both adenosine and the use of a coronary wire could cause adverse symptoms. However, the rate of potential serious adverse events is equivalent to that of routine diagnostic coronary angiography (<1%) (21). Particularly in heart transplant patients, Duran SR et al. showed no complications when using adenosine at a rate of 140 mcg/kg/min in 16 pediatric patients undergoing MR stress perfusion cardiac magnetic resonance (22). There are no studies comparing IMR with other non-invasive tools for the detection of AAR. However, rather than a substitute, IMR may be a complementary technique for rejection surveillance in HT patients. Some concerns may arise regarding the performance of an invasive microvascular function test, as it requires the use of a coronary guide and, consequently, heparin administration along with EMB extraction in the same* procedure. *However, this approach has been successfully implemented in other trials aimed at identifying CAV through intracoronary imaging techniques in HT patients, without any significant complications (23)”.*

**R2: Hypothesis: One major concern is the “softness” of the propositions used in the hypotheses. Propositions like “could reduce” or “could improve” are hardly refutable. I strongly advise to use a more direct, clearer, easy to assess hypothesis, like “The availability of IMR data in heart transplant patients will reduce the number of ECM performed during the first year … etc. Likewise, “IMR data will improve early treatment in patients with high IMR scores... etc. When the observational study is completed, these hypotheses may or may not be held right.**

R: Thank you for your suggestion. Because this is a non-randomized prospective observational study, we do not have enough consistency to use more direct language. In fact, we have observed that only one EMB was spared in the preliminary analysis of the first fifty HT patients. However, we’ve modified the text to make it clearer and more direct, here are some examples:

Using “may” instead of “could”:

- Abstract, background: *“A high IMR value early after HT may identify patients at higher risk who require increased surveillance or adjustments in immunosuppressive therapy. Conversely, a low IMR value may support reducing the number of EMBs”.*
- Discussion: *“A high IMR value early after HT may detect a higher risk patient that needs an increased surveillance or changes in immunosuppressive therapy (an earlier administration of mTor-inhibiting drugs or prescribing calcium antagonists, which are known to improve microvascular function). On the other hand, a low IMR value may lead to a decrease in the number of EMB”.*

Using “will” instead of “could”:

- Abstract, conclusions: *“IMR assessment will be useful to identify high risk heart transplant patients, leading to possible changes in management and prognosis”.*
- Hypothesis: *“High IMR baseline value will identify patients at higher risk and will lead to modifications in the number of biopsies and/or immunosuppressive therapy within one year”.*
- Discussion: *“We believe the results of this trial will be very important for our HT patient population”.*
- etc.

**R2: Experimental Groups for comparisons: Surgical/treating teams in the different Centers will be advised and informed on the protocol, enrolling 100 patients for the study. It is understandable that the physician team at every center should decide on the actual treatment applied to each patient, as stated: “Given the observational characteristics of the study, decisions regarding clinical patient management will depend on physician discretion considering patient clinical condition and other complementary tests. This, however, leads to a major problem with the proposal. The “Protocol”, states that two (or perhaps more) groups will be compared. The statistical analysis correctly described different statistical tools to be used according to the characteristics of the variable (continuous or discrete) and their distribution. What is not fully clear is how the composition of groups to be compared will be made (either a priori or a posteriori).**

R: In response to your question and as explained above: our aim when proposing a different management strategy based on IMR baseline values (<15 or ≥15) is not to compare the groups. Our goal is to observe the number of biopsies that clinicians can avoid when the IMR baseline value is low, as well as possible changes in immunosuppressive therapy when the IMR baseline value is high. We were hoping to reduce the number of biopsies that we, as interventional cardiologists, routinely perform while managing potential serious complications. If this continues the same way, a description of IMR and outcomes will therefore be presented. On the contrary, if clinicians become more “proactive” in reducing the number of biopsies according to IMR values or even in modifying immunosuppressive therapy, we’ll be able to compare between groups. Anyway, these results will be very interesting for the performance of a future randomized study in which an EMB will be spared according to IMR results.

This has been clarified in the text: page 12, lines 10-12.:

*“The number of biopsies performed by each center, as well as changes in immunosuppressive therapy, will be assessed. The results of this observational trial will lead to conduct a randomized trial* *in which an EMB will be spared based on the IMR value* ”.

**R2: According to Figure 3 of the main submission (same figure in “Protocol”), it is assumed that the main groups to be compared are patients with IMR <15 vs patients with IMR >15. Yet, each group is subdivided into “standard” and “reduced” (for EMB) or “standard” or “intensified” (for immunosuppressive therapy, IST). Since the idea is to assess the impact of knowing IRM scores to proceed on whether performing ECB, how will the groups be configured? And which group will be compared to which one? Will it be four groups in total? (i.e. IMR<15 WITH reduction in EMB; IMR<15 WITHOUT reduction in EMB; IMR>15 WITH improved/early IST; and IMR>15 WITHOUT improved/early IST)?**

R: There would be two groups in total:

- Group number one: IMR<15. See how many EMB are spared.
- Group number two: IMR≥15. See how many patients have had IST changed.

Both groups will be compared in terms of cardiac events occurrence.

We will also observe the prognostic implications of IMR variations in longer term follow up.

This has been clarified in the text, page 12, lines 7-10:

*“Both groups (IMR<15 vs IMR≥15) will be compared in terms of cardiac events occurrence. The prognostic implications of IMR variation (if that is the case) between the first three months and one year will also be analyzed”.*

**R2: Do the authors consider comparing the cases in which knowing the IMR score (above or below 15) changed the criteria for the currently/previously applied treatment?**

R: Not in this trial. We will make the comparison in the future randomized trial, when making sure changes in management are followed.

**R2: In short, how will the patients be assigned to different groups in the posterior analysis? And how do we know the number of patients where the criteria for ECB were replaced because of an IRM score >15< ?**

R: Not only is the IMR value used for decision-making, but other clinical factors (such as the clinical situation, imaging, and biomarkers) are also considered. When a center decides to spare an EMB or modify IST, it must explain which other complementary test has been considered. However, after the preliminary analysis of the first 55 patients, this is acknowledged as a limitation of the study, which is why it has been designated as a secondary objective.

**R2: Additionally, perhaps the authors consider comparing data of the enrolled patients (i.e. patients with IMR assessment from this study) with previous data of the same Centers (i.e. without IRM assessment) and then analyzing the clinical outcomes.**

R: Not in this trial. We will make the comparison in the future randomized trial, when making sure changes in management are followed.

**R2: There are discrepancies between different files composing this submission. The main file is much clearer and precise than the “Protocol” file. Although most of the text is repeated in both files, the main file does not contain important sections, such as the Hypothesis, which is found in “Protocol” and the reviewer must jump from file to file to assess the proposal.**

R: Thanks for your comments. We have corrected the discrepancies described as well as added to the manuscript the hypothesis that is present in the protocol page 6, lines 8-15: “There are few studies that evaluate IMR after a heart transplant. High IMR baseline value will identify patients at higher risk and will lead to modifications in the number of biopsies and/or immunosuppressive therapy within one year. Patients with an increase in IMR over the first year will have a higher rate of cardiac events”.

**R2: The “Protocol” file was less edited than the main file, opening concerns about Q5 on the review form (i.e. written English standards and intelligible fashion). For instance, some abbreviations are missing, and some references are presented with the same number (i.e. two works are cited as ref #10, etc.).; This file contains several grammatical mistakes, and it must be corrected, hopefully making it to coincide with the main file (references, abbreviations, definitions).**

R: We have carefully corrected all the grammatical errors you pointed out and have ensured that the texts in both the manuscript and the protocol are now consistent. We appreciate your insights, which have helped us improve the clarity and quality of our work.

**R2: The authors claim that data will be available after publication of the study, but they don’t state where these data will be available, as required by the journal.**

R: The data will be available after publication of the study at ClinicalTrials.gov**.**

**R2: In protocols, IMR is defined as: “It is calculated as the ratio of distal coronary pressure to coronary flow at hyperemia and presented in units (mmHg.s)” Although conceptually it is the ratio of pressure over coronary flow. In practice this index is calculated using the mean transit time (i.e. a reciprocal of flow; that’s why its units are mmHg x sec)). It is well explained in the main file.**

R: The definition of IMR has been standardized across all documents following the review “IMR is defined as the distal coronary pressure divided by the inverse of the hyperemic mean transit time and is expressed in units of mmHg.s”: page 11, lines 4-6.

We trust that the revisions have addressed the reviewers’ concerns and enhanced the quality of our manuscript. We remain available for further clarification or additional revisions if needed.

Thank you again for your valuable time and feedback.

Sincerely,

Ainhoa Perez Guerrero and Georgina Fuertes Ferre

Interventional Cardiology Unit

Miguel Servet University Hospital, Zaragoza
